# A Test of Levett's Patent Enamel, and Its Discovery

**Published:** 1851-04

**Authors:** C. T. Cushman

**Affiliations:** Columbus, Geo.


### ARTICLE V.

### *A Test of Levett's Patent Enamel, and its Discovery.* By C. T. CUSHMAN, D. D. S., Columbus, Geo.

"*Discover* differs from *invent.* We *discover* what before existed, though to us unknown; we *invent* what did not before exist."—*Webster's Dic.*

MR. EDITOR:—You will surely excuse a short commentary on "one of the greatest dental discoveries of the age," since, by a relation of my own experience with this so-called "new discovery," I hope to gain, in return, that of some of my professional brethren.

I have before stated, publicly, that a suitable *enamel* for covering dental plates, was still a *desideratum.* I stated this after the *modest* announcement of M. Levett had appeared, that his "philosophical and chemical composition is destined to effect a thorough and extensive change in the practice of the dental art," and before I had personally *tried* it. Having since paid $50 for the privilege of making experiments, and given them, as I think, a sufficient test, I can now say positively, that I think a suitable enamel is still a desideratum in mechanical dentistry, and, perhaps, *always will be.*

It was principally through the *unqualified* recommendations of *eminent* dentists, in various parts of the Union, which were shown me, that I was induced to purchase; and I can readily believe that the discoverer, as he admits in his circular, *is*, to an eminent degree, indebted "to his professional brethren, for their enlightened, generous and disinterested testimony."

I think it was most *too* generous, too *un*enlightened, and the "patent enamel" a substance of no practical value, notwithstanding it may have been introduced into the "most intelligent and fashionable families."

Waiving all such *substantial* claims to merit as the foregoing, or that it has a "sweetening influence on the breath," (!) I shall proceed to give the result of my own experience.

The first plate to which I applied it, after finishing and perfectly adapting it to the mouth, was one sustaining two superior incisors, in a mouth whose gums were generally prominent when speaking. I allowed the plate to turn up in front, above the teeth, and enameled this portion, in order to fill up the loss of substance of the gums, &c., and to obviate their depressed appearance. I also enameled the entire plate within the arch, and the tips of the clasps. The coating flowed well, and looked fair and comely; but, on trial in the mouth, I found the plate so *warped* by the process, (contraction of the enamel,) that it would not fit at all! In attempting to obviate this, by forcing it slightly, the enamel began to fly off in scales, as a matter of course. Notwithstanding a piece of enameled silver was exhibited by the sole agent of "exclusive rights," which he bent double and back again, repeatedly, without detriment—(perhaps this was "Japan," or other varnish,*)—enameled mouth plates will not bear the least bending or *springing* without cracking off the enamel coating.

That part of the plate in front, above the teeth, when seen upon the gum, did not appear natural, although well imitating the *color*. A thin or single coat will not sufficiently color the gold ; and more than one, on so small a surface, gives a *rounded* or cylindrical body; the line of union with the gum appears at once prominent and *artificial*, unlike a gum-tooth, which can be ground to a thin edge.

I therefore was obliged to take off the whole enameling, and for this purpose boiled the piece in muriatic acid, until the whole substance was removed; then re-adapted it. I shall, perhaps, be told, "your plate was too light, sir." I reply, it was about No. 29—not *heavy*, 'tis true, but all-sufficient for the case. It has since been daily worn, *without* enamel, nearly a year, and has never come back for alteration or adjustment.

A heavy plate enameled is *clumsy* in the mouth, and an obstruction to the tongue. Such an one, enameled on *both sides*, (as I have seen,) is intolerable and absurd. It may answer to *exhibit* to those who know nothing of the subject practically. By putting it on the gum side, the accuracy of its *fit* is defeated: it first flows into and fills all the depressions, leaving the prominences, thus *leveling* the surface in effect as if the casts were pared down.

The next case which I shall relate, was that of a temporary set for the lower jaw, on silver plate. The process of enameling was apparently satisfactory—gave a good coating and color. In less than *two months* I saw it again, and it presented a very ugly appearance. Parts of it were *scaled off*; the whole was rough and faded, as though it had been acted upon *chemically* by the juices of the mouth. However, I believe the "enamel" is not generally amenable to this charge. But it was evident, in this case, that the secretions were powerful enough to act upon it. As it then presented itself the coating was decidedly obnoxious.

* See Patent Specification, Dental Recorder, vol. ii, p. 218.

CASE 3.—An upper set on gold plate. Such very long teeth were required for this, that gum-teeth were too short to allow accurate fitting to the plate on the inside.* After enameling, this looked very well. Four months afterwards it came under my notice again—the wearer wishing to have the "stuff got off," and with some show of reason.

That part within the circle of the teeth was good; but all *without* was cracked off, *carrying with it considerable portions of the gum-teeth*, where they were ground *thin*, to fit close to the plate. Thus they were left mutilated, rough, unsightly and unpleasant to the lip; in much worse plight than before, and irremediable. This result I have observed in several other cases, and the reason appears to be this: the artificial tooth, particularly when *ground* and fitted, presents a porous surface, for which the patent enamel has a great *affinity*, and unites with it inseparably; whereas the gold plate offers no such conditions, and it by no means retains such a degree of adhesion.

The shrinking of the enamel on cooling is also unequal to that of the gold, so that every similar case, where it has been used for a *filling in* or "packing" substance, to close perfectly all joints and recesses between the teeth and plate, I apprehend it has *always* failed.

This is really the use for which, in my opinion, as I have stated in another place, a perfect enamel is still "a desideratum;" one for which Levett's patent will signally fail.

This plate, as well as the one last before mentioned, was heavier than the first; all three, indeed, sufficiently so for any similar case, neither of them springing under the hand. Nos. 28 and 29, I presume all will admit, are heavy enough, (when alloyed to twenty carats,) for most upper pieces, and No. 26 for lower.

It is not the springing or *warping* of plates alone, that wrenches it off, but the *concussion* against the artificial teeth in use, which cracks and breaks it up immediately around them—

* This is a fault that our present style of plate teeth, particularly molars for *lower* sets, will possess, until they are made at least *as long* on the lingual as on the buccal side.

as a blow upon a tree coated with ice, after a winter's rain and freezing, will fracture the glassy coating remote from the point of concussion.

In this last case, the teeth were arranged to antagonize so as to receive the lower front teeth on their cutting edge, by reason of necessity. In case No. 2, the greatest breaking up was along the molars and bicuspids, which antagonized directly with a corresponding artificial set above.

It has been objected, on the score of defence of this article, that the plates must be sufficiently thick. It is possible, therefore, that a small *horse-shoe*, enameled, might stand in the mouth, if never used in mastication. But what said the agent of patent rights to his customers, who apprehended that it might probably scale off by use? "Do you see this?" bending his enameled (query, *japanned?*) strip of silver, quite double, and then reversely double—"well, that has been done perhaps a hundred times;" and verily, it showed no detriment. But was it a fair sample? A simple coat of some kind of *varnish*, I am inclined to believe. However that may be, let no one flatter himself that *Levett's patent enamel* will stand the slightest bending of the teeth plates on which it has been vitrified; and even a few scales broken away, will make it unsightly, and exceedingly unpleasant to the sense of touch.

I have written the foregoing results of my experience, Mr. Editor, not with a view to disparage merit, nor to discourage a zealous pursuit of discovery and improvement—for I have repeatedly admitted the *desideratum*—but in hopes of getting the experience of *others* with it, through this same medium. I must believe that but few of those who recommended it, had fully and fairly tried it themselves; and that all who purchased the "exclusive right," would now be willing to sell out their stock at a large discount. For my own part, I think I would be content with ninety per cent. *off.*

I now propose to examine, briefly, the claims to "this new discovery of Dr. Levett's." Delabarre has given a process for enameling plates: Desirabode another—which he, with much candor, does not speak very highly of. It is no new thing in

France. Jewelers have enameled gold, perhaps since the days of Solomon, or since the days of glass manufacture. This kind has also been applied to dentized mouth-plates, and, (like all kinds,) as Desirabode says, pieces so enameled "do not last long."

De Loude, an English author, and old practitioner, in detailing a case of an entire dental apparatus for both jaws, which he constructed in 1836, says: "After this, I prepared the whole for enameling, (in a little muffle of a furnace,) the color of the plates corresponding with the inner side of the mouth; and to the cases that represented the back grinders, I gave a color to correspond with the teeth."*

He thus speaks of enameling as nothing *recent*, but of common practice, fourteen years ago; and only to say that he employed it in this case to obviate the "peculiar taste" which his patient experienced on wearing his former sets.

In 1834, there was a dentist in this town, Dr. H. Balsan, from Berlin, who enameled his gold plates. I have been told, by persons who have worn them, and seen them after having been worn, that the *enamel* did not stand much usage before it began to *scale off.*

* Surgical, Operative and Mechanical Dentistry, p. 167.

